# EPEPT: A web service for enhanced P-value estimation in permutation tests

**DOI:** 10.1186/1471-2105-12-411

**Published:** 2011-10-24

**Authors:** Theo A Knijnenburg, Jake Lin, Hector Rovira, John Boyle, Ilya Shmulevich

**Affiliations:** 1 Institute for Systems Biology, Seattle, WA, USA; 2 Current Address: Bioinformatics and Statistics, Division of Molecular Biology, Netherlands Cancer Institute, Amsterdam, The Netherlands

## Abstract

**Background:**

In computational biology, permutation tests have become a widely used tool to assess the statistical significance of an event under investigation. However, the common way of computing the *P*-value, which expresses the statistical significance, requires a very large number of permutations when small (and thus interesting) *P*-values are to be accurately estimated. This is computationally expensive and often infeasible. Recently, we proposed an alternative estimator, which requires far fewer permutations compared to the standard empirical approach while still reliably estimating small *P*-values [1].

**Results:**

The proposed *P*-value estimator has been enriched with additional functionalities and is made available to the general community through a public website and web service, called EPEPT. This means that the EPEPT routines can be accessed not only via a website, but also programmatically using any programming language that can interact with the web. Examples of web service clients in multiple programming languages can be downloaded. Additionally, EPEPT accepts data of various common experiment types used in computational biology. For these experiment types EPEPT first computes the permutation values and then performs the *P*-value estimation. Finally, the source code of EPEPT can be downloaded.

**Conclusions:**

Different types of users, such as biologists, bioinformaticians and software engineers, can use the method in an appropriate and simple way.

**Availability:**

http://informatics.systemsbiology.net/EPEPT/

## Background

The permutation test (also called randomization test) is a nonparametric procedure for determining statistical significance based on rearrangements of the labels of a dataset [2]. Due to its non-parametric nature, this test is commonly used in bioinformatics applications, where there is often no solid evidence or sufficient data to assume a particular model for the obtained measurements of the biological events under investigation. For example, Significance Analysis of Microarrays (SAM) [3] and Gene Set Enrichment Analysis (GSEA) [4], which detect differentially expressed genes and gene sets, respectively, are two well-known techniques that use permutation tests to compute statistical significance.

**Figure 1:**
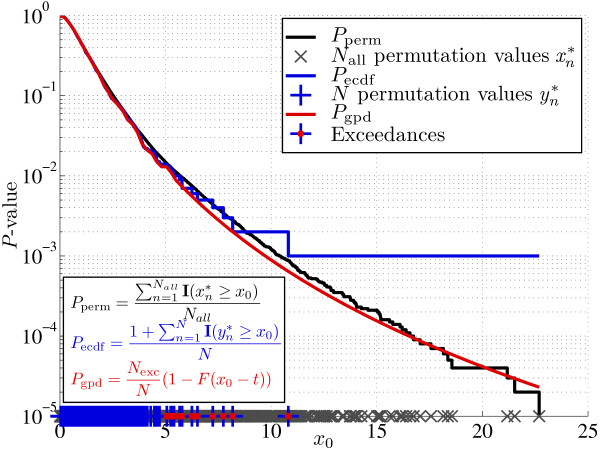
**The *P*-value of a permutation test as a function of test statistic *x*_0_**. *P*_perm _is the correct *P*-value of the permutation test based on all possible label permutations (*N*_all _= 10^5 ^in this example). The *N*_all _permutation values are visualized as gray crosses on the x-axis. *P*_ecdf _is the standard empirical estimator of the *P*-value based on a limited set of *N *permutation values (*N *= 10^3 ^in this example). These are visualized as blue plus signs on the x-axis. *P*_gpd _is the *P*-value estimator described in [1], which is also based on the *N *permutation values. It uses the 'extreme' permutation values, which exceed a particular threshold *t*. These *N*_exc _permutation values are called the exceedances and are visualized by the red circles added to the blue plus signs. In this example *t *= 5. The exceedances are used to estimate the tail of the distribution of permutation values as a generalized Pareto distribution (GPD). The GPD is represented by function *F *in the *P*_gpd _equation. From this figure it is clear that *P*_ecdf _is a poor estimator of small *P*-values, the minimum obtainable *P*-value being 1/*N*. In general, *P*_ecdf _requires 10/*P *permutations for a good estimate, *P *being the correct *P*-value. *P*_gpd_, on the other hand, provides an accurate estimate of the correct P-value, even for P-values smaller than 1/*N*.

In a permutation test, a test statistic, which is computed from the dataset, is compared with the distribution of permutation values. These permutation values are computed similarly to the test statistic, but under a random rearrangement (permutation) of the labels of the dataset. The *P*-value of a permutation test, which expresses its statistical significance, is obtained by performing all possible label permutations and computing the fraction of permutation values that are at least as extreme as the test statistic obtained from the unpermuted data. However, in practical situations, it is (by far) not feasible to perform all possible permutations. Thus, the *P*-value is typically approximated by computing a limited number of permutations, say *N*, and then computing the fraction of the *N *permutation values that are at least as extreme as the test statistic. This empirical approximation to compute the *P*-value directly couples both the minimal obtainable *P*-value and the resolution of the *P*-value to the number of permutations. Therefore, it requires a very large number of permutations when small *P*-values are to be accurately estimated. To improve upon the empirical estimator, we have employed a tail estimation procedure based on extreme value theory to estimate the tail of the distribution of permutation values and subsequently the *P*-value [1]. We showed using both theoretical and practical examples that up to several orders of magnitude fewer permutations are necessary to compute small *P*-values with the same accuracy as with the empirical approach. This results in an enormous gain in terms of computation time. For realistic datasets using the standard number of 1000 permutations, this speed-up will lead to a decrease in CPU time on the order of a couple of minutes to several hours for more complex statistics (like GSEAs running sum statistic). The approach is outlined in Figure 1 and described in detail in [1].

The aim of EPEPT is to make this approach available to the computational biology community as a general and easily accessible tool. EPEPT, which stands for **E**nhanced ***P***-value **E**stimator for **P**ermutation **T**ests, is a RESTful web API that offers dynamic programmatic access. Users submit job requests over the web either using their programming language of choice or using the website. EPEPT returns a unique URI corresponding to the submitted job. Using this URI the status of the submitted job can be checked, and upon completion, the results, i.e. the estimated *P*-values, can be retrieved.

EPEPT can be used in two different settings. In the first and most general setting, the user submits permutation values and EPEPT estimates the *P*-values, i.e. EPEPT does *not *generate the permutation statistics. In the second setting, the user submits a labeled dataset from which EPEPT first generates the permutation values and then estimates the *P*-values. Two commonly used experiment designs in computational biology are implemented: SAM and GSEA (implementation of [5]) for detection of differentially expressed genes and gene sets, respectively.

Of course, there are numerous strategies to compute permutation statistics from a labeled dataset other than the T statistic or Kolmogorov-Smirnov (KS) statistic employed in SAM and GSEA, respectively. In general, the research question and the employed dataset determine the definition of the recipe (i.e. formula) to compute permutation statistics from the labeled data set. The specification of such a recipe can be quite elaborate and complex. Forcing users to submit such a specification in a specific format/language would very much constrain the accessibility and usability of EPEPT. This is the reason why either permutation values should be submitted, or a common experiment type should be chosen. In consultation with users, additional experimental designs (based on an existing R package or their own specific data and permutation statistic, etc.) will be added to the EPEPT functionality.

## Implementation

**Figure 2:**
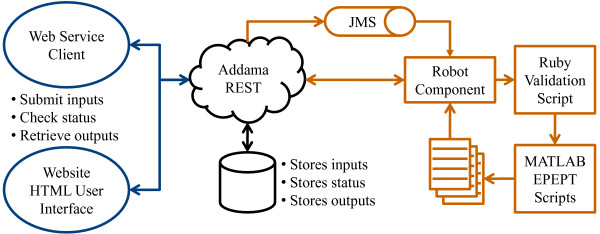
**EPEPT web service software architecture**. The user's request (either via the HTML website or programmatically) is handled by Addama, which evokes the robot component (using a JMS message) that coordinates the execution of the *P*-value estimation process. The robot communicates with Addama to provide the user access to the status, logs and outputs of the job.

Figure 2 depicts EPEPT's underlying web service software architecture. Users make an HTTP POST request that contains the input parameters describing their job. The main input is a file containing either the test statistics and their corresponding permutation values, or a labeled dataset. This depends on the setting chosen by the user. Additionally, a set of parameters, described in detail in the Usage section, can be specified. The request is handled by a RESTful Adaptive Data Management Service Architecture (Addama) [6]. After receiving the request a Java Messenging Service (JMS) message is broadcast. This message is consumed by a robot component that will coordinate the execution of the job, which is run on a separate server. First, it creates a workspace in which the inputs are stored as well as the log files, the status of the job during execution, and the produced outputs. This workspace is available to the user via a URI that is sent back after the request is made. Then, the robot component starts two scripts (in sequential order): a Ruby script for validation of the input parameters followed by the main MATLAB script. If a common experiment design is chosen (SAM or GSEA), MATLAB will call the appropriate R package (samR or GSA) to compute the permutation statistics. Based on the uploaded or computed permutation statistics, MATLAB functions are employed to perform the *P*-value estimation using the tail estimation procedure. Upon completion, the robot component persists the logs and outputs.

### Ruby validation script

The validation script checks whether all required inputs are present and populates optional parameters with default values if they are not defined by the user. Warning and error messages are logged. The script is also responsible for conversion of Excel files (.xls and .xlsx) to .csv (comma separated values) format. This conversion is necessary when the user submits an Excel file instead of a comma or tab separated text file, the latter two file types being required as input for the MATLAB scripts.

### MATLAB P-value estimation scripts

The MATLAB scripts perform the *P*-value estimation as described in Algorithm 1 of [1]. Basically, if less than ten permutation values exceed the test statistic, the estimation procedure based on tail estimation is used. Otherwise, the standard empirical procedure provides a reliable estimate and is therefore applied. (Obviously, EPEPT has been developed for the former case.)

The tail of the distribution of permutation values is modeled using a generalized Pareto distribution (GPD). Before estimating the *P*-value based on this tail approximation, a statistical test is performed to evaluate whether the exceedances of the employed test statistic can be modeled as a (GPD), (i.e. whether the distribution of the exceedances looks like a tail). This is described in the original paper in detail in [1]. If this is not the case, our approach cannot be used, and no *P*-value estimate will be returned. So far, we have not encountered statistics based on biological datasets that could not be fit with the GPD.

Two additional options have been added to the *P*-value estimation functionalities:

#### 1. Optimal order preserving transform

The tail estimation procedure is based on the exceedances, i.e. the amount by which the permutation values exceed a particular threshold *t *(see Figure 1 and [1]). These exceedances are per definition positive. Therefore, power transforms raising the exceedances (and the test statistic minus *t*) to the *n*-th power (*n *> 0) are order preserving, i.e. the order of the exceedances and the test statistic after transformation remains the same. Thus, the original formulation of the *P*-value for permutation tests, i.e. the fraction of permutation values that are at least as extreme as the test statistic, also remains the same. However, the power transform does change the weight of the tail. Power transforms with *n *> 1 will make the tail more heavy, while powers *n *< 1 will lead to a lighter tail. We have observed for both theoretical probability distributions as well as permutation value distributions based on gene expression data that, in general, larger powers will lead to a more conservative *P*-value estimate with a smaller variance [1].

When this option is selected, power transforms are applied for a range of values of *n*. The power transformation that leads to the smallest variance on the *P*-value estimate is chosen and the corresponding estimate and its variance are given. This option is recommended in cases where there is a large variance on the standard *P*-value estimate. This situation can occur when the *P*-value to be estimated is very small (e.g. on the order of 10^-9^) while using relatively few permutations (e.g. 10^3^).

#### 2. Convergence criteria

EPEPT estimates whether the *P*-value has converged or whether more permutations are necessary for a reliable estimate. Two heuristic criteria need to be satisfied for convergence:

(a) The coefficient of variation (CV) is smaller than one.

This CV is defined as follows: $\text{CV}=\frac{\frac{1}{2}\left(\log_{10}\left(P_{est}^{16}\right)-\log_{10}\left(P_{est}^{84}\right)\right)}{-\log_{10}\left(P_{est}\right)}$, where *P_est _*is the *P*-value estimate and $P_{est}^{\alpha }$ is the value of the *α*-th percentile of the estimated *P*-value. The 16th and 84th percentile values are plus/minus one standard deviation from the mean under normality assumptions. Transformation into the log_10 _domain typically makes the data more normal-like.

(b) Bootstrapped *P*-value estimates derived from all permutation values and from 10% of the permutation values should not differ in median value.

500 *P*-value estimates are derived from all permutation values using bootstrapping. Also, 500 *P*-value estimates are derived by sampling only 10% of the permutation values (with replacement). A Wilcoxon rank sum test is applied on these two sets of 500 samples to test for equal medians. If this null hypothesis is rejected with *P *< 0.001 medians are said to differ.

The convergence criteria in our original work [1] were based on the actual correct *P*-value. In practical situations EPEPT will be used to estimate the *P*-value, so the correct *P*-value is obviously not known. We have developed EPEPT's convergence criteria based on the theoretical probability distributions and gene expression datasets described in our previous work [1]. Across these data, the performance of the proposed convergence criteria (and its parameterization) was the most robust. Specifically, different criteria and different parameterizations (e.g. different percentile values) were used to call a *P*-value estimate converged or not. Of all different scenarios tested, the proposed convergence criteria agreed in most cases with the convergence criteria of our original work (based on the actual correct *P*-value, which was known for these datasets.)

### HTML User Interface

**Figure 3:**
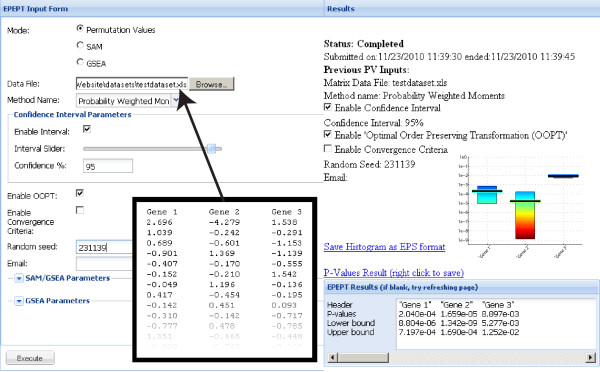
**Screenshot of the website**. In the input pane on the left all input parameters can be set. A part of the Excel file that was uploaded in this specific example is shown in the inlay (with black borders). The results pane on the right displays the execution status during execution and, on completion, displays the estimated *P*-values both numerically and graphically.

On top of this web service, a user interface was built using HTML and ExtJS, an open source cross-browser javascript library (http://www.extjs.com/). This website enables the user to upload the permutation values or labeled dataset and configure optional input parameters. Further, the results pane dynamically displays the program execution status and, on completion, visually represents the estimated *P*-values and allows the user to download them as a tab delimited text file. The results pane also displays execution time and error messages. A screenshot of this website with an example result is found in Figure 3.

## Results

This section describes the inputs that are expected by EPEPT and the logs and outputs that are generated. Also, we explain how jobs can be submitted and how the results can be obtained, using either the web service or the website.

### Inputs

• Setting of EPEPT *[required, default = PV]*

EPEPT can be run in three different settings. In the first setting ('PV'), EPEPT expects that the user uploads permutation values. In the second and the third settings, 'SAM' and 'GSEA', EPEPT assumes that a gene expression dataset is uploaded.

• File with test statistics and permutation values *or *a labeled gene expression dataset *[required] *

The file should be a tab delimited text file, a comma separated text file or an Excel file. EPEPT checks the extension of the file to decide upon its format: Excel files should have the .xls or .xlsx extension and the data should be on the first sheet. Comma separated files should have the extension .csv. All files with other extensions are assumed to be tab delimited text files.

In the 'PV' setting, each column in the file should contain one test statistic and its corresponding permutation values. Since multiple columns are allowed, different events (e.g. different genes or gene sets) can be tested simultaneously, yet independently. The file is allowed to have one header row. In case of a header row, the test statistics should be on the second row. In case no header row is used, the test statistics should be on the first row. All numerical values in the rows below the test statistic are assumed to be the permutation values. Non-numerical values, NaN's (not a number) and Inf's (infinite) are ignored. At least 1,000 permutation values per column should be reported in order for the tail estimation procedure to be used.

In the 'SAM' and 'GSEA' setting, each column should contain the expression levels of all genes in the dataset. The first row should contain the class labels or other response type assigned to the columns. Possible configurations of the first row should match the 'resp.type' options of the samR package (http://cran.r-project.org/web/packages/samr/index.html). (Also see Response Type parameter below.) The first column can be used as a header column for the gene names.

• Estimation method *[optional, default = PWM]*

Three different methods are available to estimate the parameters of the generalized Pareto distribution (which models the tail of the distribution of the permutation values): probability weighted moments (PWM), maximum likelihood (ML), and method of moments (MOM). Using theoretical distributions and practical applications we found that all methods performed comparably to each other. Some studies have been done comparing these estimators, often favoring ML [7].

• Confidence interval *[optional, default = 95]*

The confidence interval of the estimated *P*-value indicates the reliability of the estimate. The confidence interval is determined by the confidence level (default 95%). Loosely speaking, the confidence level indicates how sure (e.g. 95% sure) we can be that the actual *P*-value is within the confidence interval. This level can be set between 10 and 99.

• Confidence interval flag *[optional, default = true]*

A flag determining whether the confidence interval should be computed.

• Optimal order preserving transformation flag *[optional, default = false]*

A flag determining whether the optimal order preserving transform action should be applied.

• Convergence criteria flag *[optional, default = false]*

A flag determining whether the convergence criteria should be applied.

• Random seed *[optional, default = 0]*

If a numerical value between 1 and 1,000,000 is given, this will be used as a random seed allowing the user to reproduce EPEPT runs. When the (default) value 0 is selected, the random seed will be chosen arbitrarily.

• Email *[optional, default = empty]*

A mail will be sent to the email address (if stated) when the EPEPT run has completed. This mail contains links to the results and logs.

• Response Type *[optional, default = Two class unpaired]*

When EPEPT is used to generate permutation values in the 'SAM' or 'GSEA' setting, the user can choose the response type.

• Number of permutations *[optional, default = 1000]*

When EPEPT is used to generate permutation values in the 'SAM' or 'GSEA' setting, the user can choose the number of permutations to be performed. In the 'SAM' setting the maximum is 1,000. (SAM evaluates the *P*-value of one gene using the permutation values of all genes, effectively multiplying the number of permutations used by the number of genes.) In the 'GSEA' setting the maximum is 10,000.

• Gene set file *[required in 'GSEA' setting]*

When EPEPT is used to generate permutation values in the 'GSEA' setting, a file with gene set annotations in gene matrix transposed (.gmt) format has to be given. Such a tab delimited text file contains one gene set per row. The first two columns contain the gene set ID and description. The following columns contain the genes for that particular gene set. The annotation of these genes should match the gene annotation in the header column of the gene expression data file.

• GSEA statistic *[optional, default = maxmean]*

When EPEPT is used to generate permutation values in the 'GSEA' setting, the user can choose the statistic used to summarize genesets (see [5] and http://cran.r-project.org/web/packages/GSA/index.html).

### Logs

During the execution of the job, Addama, Ruby and MATLAB are creating log files containing standard output, warnings and errors, which are made available to the user.

### Outputs

The main output of EPEPT is the set of estimated *P*-values. These are reported in a tab delimited text file. If headers were provided in the original file, the output file contains the same headers. If confidence intervals were requested the two rows under the row with the *P*-value estimates indicate the lower and upper bound of the confidence intervals. Finally, if the convergence criteria were applied another row is added with binary values indicating whether the estimate converged (1) or not (0).

Besides this text file, two picture files (a .png and an .eps file) are generated that visually depict the estimated *P*-values and their confidence bounds.

#### Web service

EPEPT is web service enabled which means that EPEPT can be accessed programmatically via any programming language with HTTP support, such as C, Java, MATLAB, Perl, Ruby, R, etc. The programmatic flow to make a request to the EPEPT processing host is as follows:

1. The user (i.e. web service client) initializes the set of input parameters and sets them to the user-defined values.

2. The client makes a POST request with the input parameters to the EPEPT host. A unique URI is returned.

3. The client checks the status of the submitted request using the unique URI. The status can be: RUNNING, COMPLETED or ERROR. The client program will loop until the status is COMPLETED or ERROR.

4. The client retrieves the output and/or log files from the host and stores these locally.

In summary, after the request has been made, everything (concerning the client) evolves around the assigned URI. The inputs, logs and outputs are accessible via uri/inputs/, uri/logs/and uri/outputs/, respectively, where uri is the URI assigned to the user by EPEPT.

**Figure 4:**
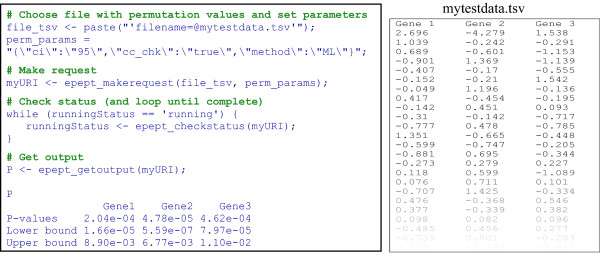
**Example R code to run EPEPT**. The inputs (including the tab delimited text file 'mytestdata.tsv' that contains the permutation values) are submitted by making a request to EPEPT after which a unique URI is returned to the client. Using this URI the status of the submitted job can be checked. When the job is completed, the estimated *P*-values are retrieved using the URI and stored in variable P.

Figure 4 presents a small example, where R is employed to run EPEPT. The EPEPT website http://informatics.systemsbiology.net/EPEPT/ provides examples for four programming languages (R, Perl, MATLAB and Ruby) and offers downloads to the libraries necessary to run these examples. Also, test data sets and documentation on the exact input requirements (i.e. the variable names to be used) are available.

#### Website

The website is a simple HTML input form, where the file with the permutation values or labeled dataset can be uploaded and all options can be set using sliders, drop down menus, check boxes and text fields. The results are presented back to the user in the results pane, which depicts the estimated *P*-values both as text as well as graphically and gives download links for these output files. See Figure 3.

A manual for the HTML input form is hosted on the EPEPT Google Code project http://code.google.com/p/epept/. In addition to the manual, this site also hosts the source code, examples datasets and web service client examples. Links to the EPEPT Google Code website are found on the EPEPT website.

#### Source code

The latest version of the source code for the complete EPEPT package is available for download from the EPEPT Google Code project http://code.google.com/p/epept/. A stand alone version of EPEPT for MATLAB is also included.

## Conclusions

Due to the enormous increase in biological data and in the computational complexity of its analysis, computational biology is shifting towards client-server based computing models. In these models computational analysis is no longer performed on the desktop, but tasks are farmed out to different (web-based) service providers, such as network clusters, web services or cloud computing environments. Indeed, besides programmatic access to databases, programmatic access is also becoming available for more and more bioinformatics tools [8].

Here, we have presented EPEPT, a web service tool to estimate *P*-values for permutation tests based on extreme value theory. The EPEPT estimator forms a valuable alternative to the standard empirical estimator, since it can provide accurate *P*-value estimates in (the frequently occurring) situations, where no or only very few permutations values exceed the test statistic even when a considerable number of permutations have been performed. The programmatic access to these routines is practical for computational biologists that aim to systematically test many hypotheses (e.g. many genes or gene sets) using the programming language of their choice. Moreover, EPEPT can easily be integrated into automatic workflows.

At this moment, EPEPT uses a Linux server (4 cores, 8 processors, 3.00 GHz, 32 Gbs RAM) for computation (and another server as web/tomcat server). However, the web application and computation used for EPEPT can and will be deployed as cloud computing services, if scaling up is necessary.

Because EPEPT utilizes the Addama Service Architecture [6], the core EPEPT service executes outside the web server domain. Consequently, this design promotes rapid development, debugging and deployment. That is, bug fixes, updates and additional functionalities can be implemented by simply adapting the MATLAB *P*-value estimation scripts, which are then immediately available both via the website and via programmatic access. One future enhancement will be to allow for URI file access, such that clients can provide the URI of the file with permutation values in stead of sending the complete file when making a job request. Additionally, in the future EPEPT will provide the client with the estimated time left before the job is completed both via a progress bar on the website and via the status check using the job-specific URI.

## Availability and requirements

Project name: Enhanced *P*-value Estimator for Permutation Tests (EPEPT)

Project home page: http://informatics.systemsbiology.net/EPEPT/

Operating system(s): Platform independent

Programming language: any language that can interact with the web

License: GNU Lesser General Public License (LGPL)

## Abbreviations

API: Application Programming Interface; EPEPT: Enhanced *P*-value Estimator for Permutation Tests; HTTP: Hypertext Transfer Protocol; REST: Representational State Transfer; URI: Uniform Resource Identifier

## Authors' contributions

TAK implemented the MATLAB *P*-value estimation scripts and MATLAB web service client example. JL implemented the Ruby validation script, the robot component, the HTML website and the other web service clients examples. HR designed the robot architecture and assisted with integration of EPEPT into the Addama Service Architecture. TAK and JL drafted the manuscript. JB and IS supervised the project. All authors have read and approved the final manuscript.
